# Anti-angiogenic Agents in Combination With Immune Checkpoint Inhibitors: A Promising Strategy for Cancer Treatment

**DOI:** 10.3389/fimmu.2020.01956

**Published:** 2020-08-25

**Authors:** Yuxiao Song, Yang Fu, Qi Xie, Bo Zhu, Jun Wang, Bicheng Zhang

**Affiliations:** ^1^Cancer Center, Hubei Provincial Research Center for Precision Medicine of Cancer, Renmin Hospital of Wuhan University, Wuhan, China; ^2^Department of Oncology, Xiangyang Hospital, Hubei University of Chinese Medicine, Xiangyang, China; ^3^Medical Research Centre, The First Affiliated Hospital of Shandong First Medical University, Jinan, China; ^4^Institute of Cancer, Xinqiao Hospital, Army Medical University, Chongqing, China; ^5^Department of Oncology, The First Affiliated Hospital of Shandong First Medical University, Jinan, China

**Keywords:** immune checkpoint inhibitor, PD-1, PD-L1, anti-angiogenesis, tumor microenvironment, combination therapy

## Abstract

Advances in cancer immunity have promoted a major breakthrough in the field of cancer therapy. This is mainly associated with the successful development of immune checkpoint inhibitors (ICIs) for multiple types of human tumors. Blockade with different ICIs, including programmed cell death 1 (PD-1), programmed cell death-ligand 1 (PD-L1), and cytotoxic T-lymphocyte-associated protein 4 (CTLA-4) inhibitors, may activate the immune system of the host against malignant cells. However, only a subgroup of patients with cancer would benefit from immune checkpoint blockade. Some patients experience primary resistance to initial immunotherapy, and a majority eventually develop acquired resistance to ICIs. However, the mechanisms involved in the development of drug resistance to immune checkpoint blockade remain unclear. Recent studies supported that combination of ICIs and anti-angiogenic agents could be a promising therapeutic strategy for overcoming the low efficacy of ICIs. Moreover, through their direct anti-cancer effect by inhibiting tumor growth and metastasis, anti-angiogenic drugs reprogram the tumor milieu from an immunosuppressive to an immune permissive microenvironment. Activated immunity by immune checkpoint blockade also facilitates anti-angiogenesis by downregulating the expression of vascular endothelial growth factor and alleviating hypoxia condition. Many clinical trials showed an improved anti-cancer efficacy and prolonged survival following the addition of anti-angiogenic agents to ICIs. This review summarizes the current understanding and clinical development of combination therapy with immune checkpoint blockade and anti-angiogenic strategy.

## Introduction

Cancer immunotherapy has achieved great advances during the past several decades due to improved understanding of tumor immunobiology, which boost durable tumor immune response, lead to long-term tumor remission, and even cure a subgroup of patients (1). Prominent immunotherapy involves the use of immune checkpoint inhibitors (ICIs) targeting negative regulators of specific CD8+ T cells or immune checkpoints, such as programmed cell death 1 (PD-1), programmed cell death ligand 1 (PD-L1), and cytotoxic T-lymphocyte-associated protein 4 (CTLA-4) (2). Ipilimumab is the first antagonistic CTLA-4 antibody approved by the US Food and Drug Administration in 2011, improving overall survival (OS) in patients with advanced or metastatic melanoma. Monotherapy with other antibodies targeting PD-1 or PD-L1 has been shown to be effective against numerous types of human tumors (3). Furthermore, promising results from clinical trials inspired further investigations regarding the possible blockade of alternative immune checkpoints. However, only a proportion of patients are initially responsive to immune checkpoint blockade and a majority are intrinsically resistant to these therapies, partly because of the low number of infiltrating lymphocytes in the tumor bed (4). In addition, most patients eventually develop acquired resistance to ICIs, and the mechanisms involved in the development of drug resistance remain unknown (2).

Anti-angiogenesis is another promising therapeutic strategy designed to disrupt the vascular supply and starve tumor of nutrients and oxygen. This is achieved mainly by blocking the vascular endothelial growth factor (VEGF)/VEGF receptor (VEGFR) signaling pathway that is active in the tumor microenvironment with hypoxia condition. As a result, there is a local balance between pro-angiogenic factors, anti-angiogenic factors, and vessel normalization following anti-angiogenic therapy (5). A total of 11 anti-angiogenic agents, including anti-VEGF antibody, anti-VEGFR antibody, as well as VEGFR tyrosine kinase inhibitors (TKIs), have been approved for certain types of cancer. However, monotherapy with an anti-angiogenic agent or combination with chemotherapy or targeted therapies demonstrated limited clinical benefits for most patients with cancer. Given that both anti-angiogenesis and immune checkpoint blockade focus on targeting the tumor microenvironment, the combination of ICIs and anti-angiogenic agents presents a potential synergistic anti-tumor effect (6). This review summarizes the current understanding and clinical development of combination therapy of immune checkpoint blockade and anti-angiogenesis.

## Synergistic Anti-Tumor Mechanisms Attributable to Anti-Angiogenic Agents in Combination With ICIs

It is well-established that tumor angiogenesis is deregulated under the continuous stimulation of excessive release of pro-angiogenic factors, such as VEGF, which can be induced by hypoxia or specific genetic alterations. Junctional defects of endothelial cells result in leaky tumor vasculature that is usually tortuous, dilated, and poorly covered with pericytes. Anti-angiogenic therapy can downregulate continuous angiogenic signaling and result in vasculature normalization, such as pruning, vasculature maturation, and increased perfusion (5). As a result, the local concentration and efficacy of other anti-cancer drugs, including cytotoxic drugs, targeted drugs, and ICIs, are significantly improved (7).

The cancer immunity cycle described by Chen and Mellman (8) includes seven important steps, which may control an effective and systematic anti-tumor immune response: (a) release of cancer cell antigens; (b) cancer antigen presentation; (c) priming and activation of antigen-presenting cells and T cells; (d) trafficking of T cells to tumors; (e) infiltration of T cells into tumors; (f) recognition of cancer cells; and (g) killing of cancer cells. In fact, VEGF signaling decreases the anti-tumor response by influencing multiple steps of the cancer immunity cycle (9), including the functional maturation of dendritic cells (DCs), priming and activation of T cells, trafficking of T cells from the lymph node to the tumor bed, and infiltration of T cells into tumors. Thus, anti-angiogenic drugs could recover the host's potent anti-tumor immune response by interfering with the multiple steps of the cancer immunity cycle.

### Anti-angiogenic Agents Promote Antigen Presentation and Activate Cytotoxic CD8+ T Cells

DCs are the main antigen-presenting cells and derived from hematopoietic bone marrow prognostic cells. Immature DCs fail to present cancer antigens to T cells because of the lack of expression of surface MHC-I, MHC-II, and costimulatory molecules. Mature DCs have been found to be inversely correlated with increased VEGF levels in the circulation (10); however, patients with immature DCs in the peripheral blood had increased levels of plasma VEGF. VEGF affects the differentiation and maturation of DCs by inhibiting the transcriptional activation of nuclear factor-κB (11), resulting in inactivation of cytotoxic T lymphocytes. It was found that VEGF blockade results in a more mature DC phenotype in mouse models of glioblastoma, demonstrated by the increasing expression of the co-stimulatory molecules B7-1, B7-2, and MHC-II (12). Specifically, anti-angiogenic agents alleviate the restraining effects of VEGF on the migration capacity and immune function of DCs (13). Thus, normalization of the tumor vasculature promotes antitumor immunity by enhancing the uptake of antigen presentation in DCs.

Under high concentrations, VEGF induces apoptotic pathways in cytotoxic CD8+ T cells by increasing the expression of PD-1 and activates regulatory T cells (Tregs) in a VEGFR2-dependent manner (14). Activation of the PD-1/PD-L1 pathway regulates CD4+ T-cell differentiation into Tregs expressing the forkhead box P3 (FOXP3), directly triggering immune suppression. Mulligan et al. (15) have shown that, in oral squamous carcinoma, VEGF induces the secretion of prostaglandin E2 (PGE2) by endothelial cells. PGE2 is an immune suppressant disrupting T-cell activation and suppressing T-cell generation and cytotoxic functions (15). In addition, there are results showing that memory CD4+ T cells can recognize neoantigens and may lead to cancer remission after immunomodulation in the hepatocellular carcinoma (HCC) microenvironment after treatment with sorafenib (16). Meanwhile, other findings imply that stable vessels due to treatment with bevacizumab in pancreatic ductal adenocarcinoma supply anti-cancer immune cells including memory CD4+ T cells, which are at least partially responsible for the better OS observed in patients with tumors expressing high levels of CD31 (17).

### Anti-angiogenic Agents Promote the Infiltration and Migration of Lymphocytes

The migration of lymphocytes from blood to the tumor stroma is affected by the integrity of the tumor vasculature. Anti-angiogenic antibodies, such as VEGF inhibitors (e.g., bevacizumab), can promote T-cell infiltration into solid tumors and enhance the efficacy of immunotherapy (18). Additionally, a mouse model using a BRAF inhibitor in combination with adoptive transfer of T cells revealed that the downregulation of VEGF was responsible for the increase in T-cell infiltration (19). Anti-angiogenic agents suppress tumor growth and restore microvessel density, as well as upregulate endothelial adhesion molecules in tumor vessels which lead to enhanced T-cell infiltration. Dirkx et al. (20) discovered that in two mouse models (i.e., colon carcinoma LS174T/nude mice and B16F10 melanoma/C57bl/6), treatment with angiogenesis inhibitors (i.e., anginex, endostatin, angiostatin, and paclitaxel) improved the leucocyte-vessel wall interaction by upregulation of adhesion molecules.

The angiopoietins affect inflammation and immune trafficking by increasing the expression of platelet endothelial cell adhesion molecule-1 (PECAM-1) and vascular endothelial-cadherin, and decreasing that of vascular-cell adhesion molecule-1 (VCAM-1), intracellular adhesion molecule-1 (ICAM-1), and endothelial leukocyte adhesion molecule 1 (ELAM-1) (21). Consequently, inhibition of angiopoietins or other angiogenic activators may restore normal immune cells trafficking and promote lymphocyte infiltration into the tumor (22). Pro-angiogenic factors in the tumor microenvironment suppress the expression of adhesion molecules and chemokines, such as CXC chemokine ligands 10 (CXCL10) and CXCL11, that attract cytolytic T cells and natural killer cells (23). Nitric oxide and epidermal growth factor-like domain 7 (EGFL7) can also regulate the expression of adhesion molecules in tumors (24, 25). Bevacizumab in combination with atezolizumab increases the number of intra-tumor CD8+ T cells, and increases the expression of intra-tumor chemokine CX3C-ligand1 (CX3CL1) and the CX3CL1 receptor (CX3CR1) on CD8+ T cells (26) and trafficking lymphocytes in metastatic renal cell carcinoma (mRCC) models. These findings suggest that this combination treatment improves antigen-specific T-cell migration.

### Anti-angiogenic Agents Reduce Immunosuppression

Immunosuppression acts as a survival mechanism of cancer cells to escape elimination by the human immune defense system. Cancer cells can inhibit the generation of anti-tumor response through a variety of immunosuppression-related mechanisms, such as downregulating the expression of antigens and MHC-I, altering the immunosuppressive signal, secreting inhibitory factors that dampen the activity of immune cells, and recruiting inhibitory immune cells (27, 28). Moreover, endothelial cells in the tumor vasculature also control immunosuppression by modulating the activity and variability of immune cells. Firstly, the Fas ligand is upregulated in response to tumor-derived VEGF, interleukin 10 (IL-10), and PGE3. Secondly, some inhibitory molecules, including PD-L1, T cells immunoglobulin, and domain-containing protein 3 (TIM3), tumor necrosis factor-related apoptosis-inducing ligand, and transforming growth factor-β (TGF-β) are found in endothelial cells. This suggests a possible regulatory effect of endothelial cells on angiogenesis (29). Thus, anti-angiogenic therapy also regulates immunosuppression by changing the function of tumor vasculature endothelial cells. Compelling evidence indicates that myeloid-derived suppressor cells (MDSCs), Tregs, tumor-associated macrophages (TAMs), regulatory DCs, neutrophils, T helper 17 (Th17) cells, and regulatory B cells are key immunosuppressive cells that promote tumor progression (30, 31). The immunosuppressive tumor microenvironment is established by recruiting immunosuppressive cells and tumor cell-derived immunosuppressive cytokines, such as VEGF, TGF-β, galectin, or indoleamine 2-3-dioxygenase (32). MDSCs, TAMs, and Tregs are the major effector cells in this immunosuppressive microenvironment. MDSCs can diminish the antitumor immune response in such a microenvironment by: (a) inducing Tregs; (b) producing immunosuppressive cytokines, such as TGF-β; (c) depleting or sequestering the amino acids arginine, tryptophan, or cysteine required for the function of T cells; or (d) nitrating the T-cell receptor or chemokine receptors on tumor-specific T cells (33). Meanwhile, tumor-derived granulocyte-macrophage colony-stimulating factor, IL-1β, VEGF, and PGE2 lead to the accumulation of MDSCs in the tumor microenvironment (33).

Normalization of the tumor vasculature can reduce the immunosuppression exerted by Tregs, TAMs, and MDSCs (34). Increases in intratumoral CD8+ T cells and macrophages were observed after monotherapy with bevacizumab in mRCC. These effects resulted in an upregulation of MHC-I on tumor cells, and increased the expression of Th1 and T effector gene signatures in post-dose biopsies (35).

Tregs can secrete the immunosuppressive cytokine IL-10, TGF-β, and express immune checkpoint molecules (e.g., PD-1) to inhibit the activity of antigen-presenting cells and effector cells. However, the reduced induction of T-cell differentiation into Tregs and promoting effector Th cells, which generate interferon-γ (IFN-γ), can be implemented by blocking the signal transducer and activator of transcription 3 (STAT3) signaling pathway, thereby suppressing tumor growth (36). CXC chemokines are potent angiogenic factors promoting the migration and generation of endothelial cells (37). Anti-angiogenic therapy targeting VEGF/VEGFR may restrain the expression of CXCL1, IL-1β, and IL-6 to inhibit Tregs chemotaxis and accumulation in tumors (38).

Alleviated hypoxia, owing to normalization of the tumor vasculature, preferentially induces polarization of TAMs to the M1-like phenotype (39). Tumor cells secrete VEGF-A and VEGFR2 to improve the formation and effectiveness of MDSCs (40), which can be reduced by some anti-angiogenic drugs. This indicates the potential for utilization, with an indirect impact of MDSCs on alterations in Tregs. Overall, anti-angiogenic therapy reprograms the tumor vasculature by normalization and leads to improved anti-tumor immune response.

### ICIs Can Enhance the Anti-cancer Effects of Anti-angiogenic Agents by Relieving Immunosuppression

In addition to eliciting immune-mediated elimination of tumor cells, ICIs were also demonstrated to promote normalization of the tumor vasculature in orthotopic breast and ectopic colon tumor models (34, 41). In both studies, blockade of CTLA-4 or PD-1 reduced tumor vascular density, improved vessel perfusion, and alleviated tumor tissue hypoxia, all of which were marks of the vascular normalization effect. In mouse models lacking CD4+ T lymphocytes (CD4–/–) or CD8 T lymphocytes (CD8–/–) through genetic knockout, it was found that blockade of CTLA-4 and PD-1 facilitated tumor vessel normalization by the activation of CD4+ Th1 cells (34). Furthermore, the exhaustion of Tregs led to aggregation of CD8+ effector T cells and increased the production of IFN-γ, along with increased tumor vessel perfusion detectable by Doppler ultrasonography prior to tumor shrinkage (41). Thus, it is suggested that the vasculature-normalizing effect of ICIs is mainly mediated by the activation of CD8+ T cells via the IFN-γ signaling pathway. IFN-γ elevated the expression of endothelial adhesion molecules ICAM1 and e-selectin, which mediate immunocyte infiltration, decreased that of endothelial VEGFA and increased the expression of CXCL9, CXCL10, and CXCL1, which recruit Th1 cells (41). In addition, Th1 type chemokines (e.g., CXCL9 and CXCL10) can engender angiostatic effects (42) by stimulating the recruitment of pericytes (41), apart from acting as chemoattractants for effector T cells (43). Interestingly, the decrease of Tregs also stimulates the infiltration of eosinophils in tumors (44). In addition, anti-CTLA-4 therapy has been reported to increase the infiltration of eosinophils via the activation of memory CD4+ and CD8+ T lymphocytes in breast tumor models (45). Meanwhile, IFN-γ is secreted to promote tumor vessel normalization, enhancing the infiltration of effector T cells.

**Figure 1:**
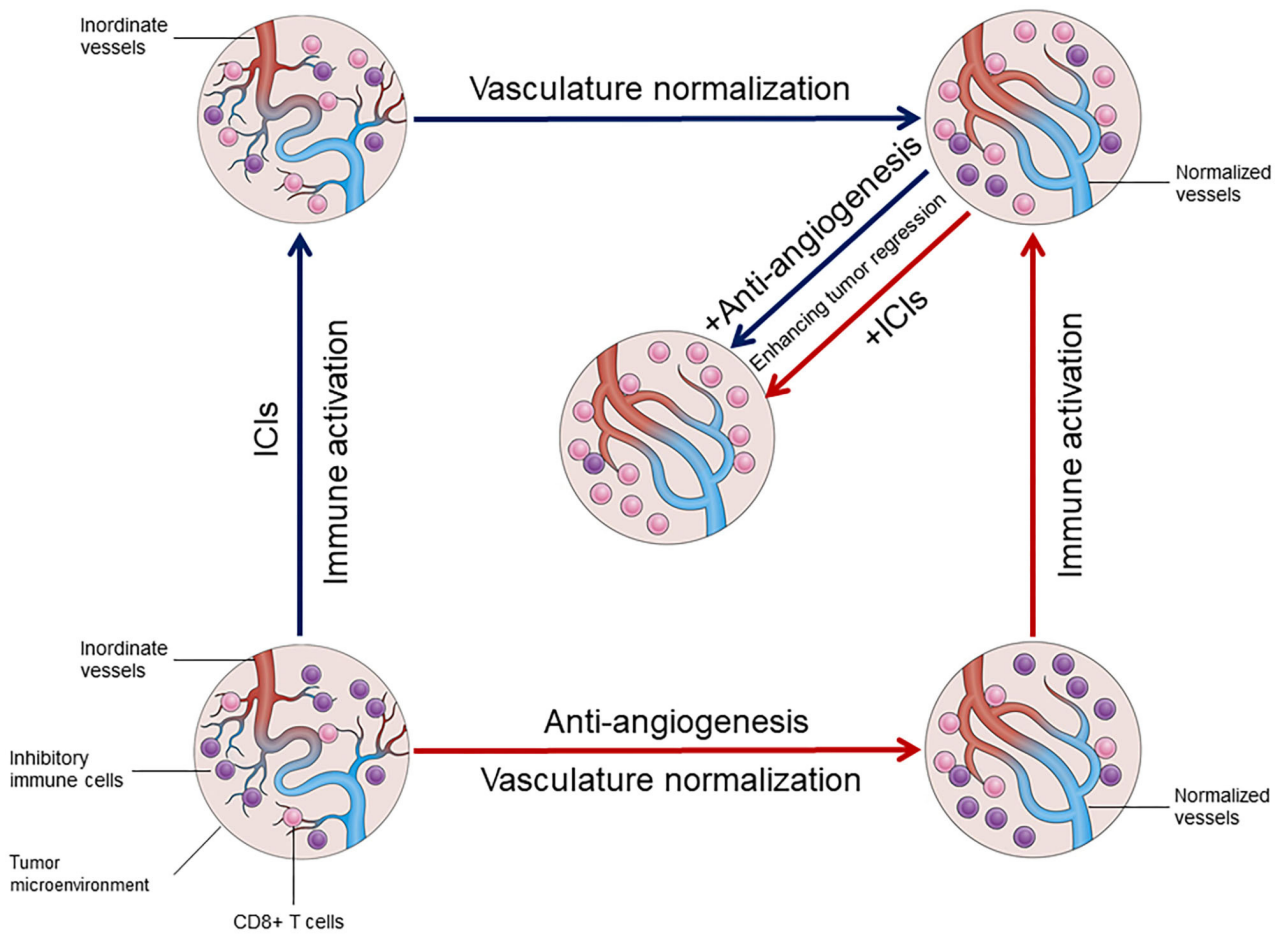
The mutual regulation between vasculature normalization and immune activation in tumor microenvironment.

Immune checkpoints PD-1/PD-L1 play an important role in immunosuppression in various types of cancer. Some inhibitory cells, including MDSCs, Tregs, and M2-macrophages, as well as secretory immunomodulatory factors are pivotal for immunosuppression (46). In fact, these inhibitory cells also stimulate angiogenesis by increasing the expression of pro-angiogenic factors from the extracellular matrix. The immunosuppressive tumor microenvironment attributed to repressive inflammatory cells may play a major role in mediating the adaptive resistance to anti-angiogenic agents. It is well-known that anti-VEGF therapy can cause tumor hypoxia due to excessive vessel regression, M2-TAMs attracted by oncostatin M and eotaxin from hypoxic tumor cells might be a compensatory mechanism to secure tumor angiogenesis through providing pro-angiogenic factors (47), which seems partially responsible for the resistance to anti-VEGF therapy. Furthermore, MDSC-derived Bv8 (prokinectin 2) is found to directly promote tumor angiogenesis even when the VEGF signaling pathway is blocked (48, 49). These studies have provided compelling evidence that anti-angiogenic therapy is more effective following the generation of an immunostimulatory microenvironment. Based on various studies, the proposition that angiogenesis and inflammation are mutually regulated has been accepted (50–52). As mentioned, alleviated hypoxia and normalization of the tumor vasculature by ICIs also contribute to reprogram the immunosuppression environment, which in turn enhances the anti-tumor effects of both therapies. Thus, alleviated immunosuppression coupled with normalization of the tumor vasculature eventually achieve a loop of positive feedback that promotes each other (Figure 1). This further assists in identifying new, non-invasive, predictive biomarkers for immunotherapy and develop more effective combination strategies. Functional detection of vascular remodeling within the tumor microenvironment with Doppler ultrasonography, perfusion scans, or dynamic contrast-enhanced magnetic resonance imaging can more accurately represent tumor vascular rebuilding induced by ICIs, which extends beyond the traditional tumor cell and immune cell analysis (43). With the normalization of the tumor vessels and tissue perfusion as well as the promotion of oxygenation, ICIs therapy may increase the concentration of agents from other systemic therapies in tumors (53) and enhance radiosensitivity. Nevertheless, more detailed investigation is warranted to delicately investigate the feasibility of the new ICIs combination strategies.

## Clinical Data Involving the Combination of Anti-Angiogenic Agents and ICIs

Numerous clinical trials and other studies have focused on the combination of anti-angiogenic agents and ICIs. The results of these clinical trials are displayed in Table 1. Table 2 exhibits the currently ongoing or recruiting clinical trials to investigate the efficacy of ICIs plus anti-angiogenic agents.

**Table 1:** Clinical trials investigating the combination effect of anti-angiogenic agents and ICIs.

**Clinical trials**	**Phase**	**Cancer type**	**Anti-angiogenic agents**	**ICI**	**Results**	**AEs (total, Grade 3–5)**
NCT02366143 (54) (IMpower150)	III	NSCLC	Bevacizumab	Atezolizumab	PFS: 8.3 m OS: 19.3 m	Total = 94% Gr 3–4: 223 (57%) Gr 5: 11 (3%)
NCT02039674 (55) (KEYNOTE-021)	I	NSCLC	Bevacizumab	Pembrolizumab	PFS: NR ORR: 56%	Total = 96% Gr 3–4: 10 (42%)
NCT01454102 (56)	I	NSCLC	Bevacizumab	Nivolumab	PFS: 37.1 w	Total = 92% Gr 3: 4 (33.3%)
NCT02443324 (57)	I	NSCLC	Ramucirumab	Pembrolizumab	PFS: NR ORR: 30%	Total = 81% Gr 3–4: 2 (7%)
NCT03359018 (58)	NA	NSCLC	Anlotinib	Sintilimab	DCR: 100% ORR: 72.7%	Total = NA Gr 3–4: 6 (27.3%)
NCT00790010 (59)	I	Melanoma	Bevacizumab	Ipilimumab	DCR: 67.4% OS: 25.1 m	Total = 100% Gr 3: 11 (23.9%) Gr 4: 2 (4.3%)
NCT03722875 (60)	NA	HCC	Apatinib	Camrelizumab	ORR: 30.8%	Total = NA Gr ≥3: 20 (60.6%)
NCT02715531 (61)	I	HCC	Bevacizumab	Atezolizumab	PFS: NR OS: NR	Total = 81% Gr 3–4: 9 (35%)
NCT03434379 (62) (IMbrave 150)	III	HCC	Bevacizumab	Atezolizumab	PFS: 6.8 m ORR: 27%	Total = 84% Gr 3–4: 117 (36%) Gr 5: 6 (2%)
NCT03006926 (63)	Ib	HCC	Lenvatinib	Pembrolizumab	PFS: 8.6 m OS: 22.0 m	Total = 95% Gr ≥3: 67 (67%) Gr ≥4: 4 (4%)
NCT02443324 (64)	I	GC/GEJ	Ramucirumab	Pembrolizumab	PFS: 2.1 m/2.6 m	Total = 78% Gr 3–4: 10 (25%)
NCT02572687 (65)	I	GC/GEJ	Ramucirumab	Durvalumab	PFS: 2.6 m ORR: 36%	Total = 83% Gr 3: 10 (35%)
NCT03475953 (66) (REGOMUNE)	II	CRC	Regorafenib	Avelumab	PFS: 3.6 m OS: 10.8 m	Total = NA Gr 3–4: 32 (66%)
NCT02420821 (67) (IMmotion151)	III	RCC	Bevacizumab	Atezolizumab	PFS: 11.2 w	Total = NA Gr 3–4:182 (40%)
NCT02493751 (68)	I	RCC	Axitinib	Avelumab	OS: 58%	Total = NA Gr ≥3: 32 (58%)
NCT02684006 (69) (JAVELIN Renal 101)	III	RCC	Axitinib	Avelumab	PFS: 16.6 m ORR: 60.6%	Total = 64% Gr ≥3: 20 (30%)
NCT02501096 (70)	II	RCC	Lenvatinib	Pembrolizumab	PFS: 17.7 m ORR: 66.7%	Total = NA Gr 3–4: 21 (70%)
NCT02853331 (71) (KEYNOTE-426)	III	RCC	Axitinib	Pembrolizumab	PFS: 15.1 m ORR: 59.3%	Total = NA Gr ≥3:327 (75.8%)
NCT01472081 (72) (CheckMate 016)	NA	RCC	Sunitinib	Nivolumab	PFS: 12.7 m ORR: 55%	Total = 100% Gr 3–4: 27 (82%)
NCT02501096 (73)	II	EC	Lenvatinib	Pembrolizumab	PFS: 7.4 m ORR: 39.6%	Total = NA Gr 3: 31 (59%)

*The details were obtained from http://clinicaltrials.gov/*.

*m, months; w, weeks; EC, endometrial cancer; GC, gastric cancer; GEJ, gastroesophageal junction adenocarcinoma; HCC, hepatocellular carcinoma; NSCLC, non-small cell lung cancer; RCC, renal cell cancer; NA, not applicable; NR, not reached; Gr, grade*.

**Table 2:** Ongoing clinical trials investigating the efficacy of ICIs plus anti-angiogenic agents.

**Trial identifier**	**Disease**	**Treatment (arm of combination therapy)**	**Phase**	**Status**
NCT03024437	RCC	Atezolizumab + bevacizumab + entinostat	I/II	Recruiting
NCT03363867	OC	Atezolizumab + bevacizumab + cobimetinib	II	Recruiting
NCT03472560	NSCLC/UC	Avelumab + axitinib	II	Recruiting
NCT03395899	BC	Atezolizumab + bevacizumab + cobimetinib, neoadjuvant	II	Recruiting
NCT02724878	NCCKC	Atezolizumab + bevacizumab	II	Recruiting
NCT03386929	NSCLC	Avelumab + axitinib + palbociclib	I/II	Recruiting
NCT03574779	OC	TSR-042 + bevacizumab + Niraparib	II	Recruiting
NCT02921269	CC	Atezolizumab + bevacizumab	II	Active, not recruiting
NCT03647956	NSCLC	Atezolizumab + bevacizumab + carboplatin + pemetrexed	II	Recruiting
NCT02734004	OC/BC/SCLC/GC	MEDI4736 + bevacizumab + olaparib	I/II	Recruiting
NCT03517449	EC	Pembrolizumab + lenvatinib	III	Recruiting
NCT02572687	GC/GEJ/NSCLC/HCC	MEDI4736 + ramucirumab	I	Active, not recruiting
NCT02839707	OC/FTC/PC	Atezolizumab + bevacizumab + PLD	II/III	Recruiting
NCT02210117	RCC	Ipilimumab + bevacizumab, neoadjuvant	I	Active, not recruiting
NCT01950390	Melanoma	Ipilimumab + bevacizumab	II	Active, not recruiting
NCT03394287	BC	Camrelizumab + apatinib	II	Recruiting
NCT03417895	SCLC	Camrelizumab + apatinib	II	Not yet recruiting
NCT03491631	Multiple solid tumors	Camrelizumab + apatinib + SHR9146	I	Not yet recruiting
NCT02942329	HCC/GC	Camrelizumab + apatinib	I/II	Recruiting
NCT03671265	ESCC	Camrelizumab + apatinib + radiation	NA	Not yet recruiting
NCT03359018	Osteosarcoma	Camrelizumab + apatinib	II	Active, not recruiting
NCT03755791	HCC	Atezolizumab + cabozantinib	III	Recruiting
NCT03502746	Mesothelioma	Nivolumab + ramucirumab	II	Recruiting
NCT03606174	UC	Nivolumab + sitravatinib	II	Recruiting
NCT03680521	RCC	Nivolumab + sitravatinib, neoadjuvant	II	Recruiting
NCT02493751	RCC	Avelumab + axitinib	I	Active, not recruiting
NCT01633970	Multiple solid tumors	Atezolizumab + bevacizumab	I	Active, not recruiting

*The details were obtained from http://clinicaltrials.gov/. Trials without “neoadjuvant” are focusing on metastatic or advanced settings*.

*BC, breast cancer; CC, cervical cancer; EC, endometrial cancer; ESCC, esophageal squamous cell carcinoma; FTC, fallopian tube cancer; GC, gastric cancer; GEJ, gastroesophageal junction adenocarcinoma; HCC, hepatocellular carcinoma; NA, not applicable; NCCKC, non-clear cell kidney cancer; NSCLC, non-small cell lung cancer; OC, recurrent ovarian cancer; PC, peritoneal cancer; RCC, renal cell cancer; SCLC, small cell lung cancer; UC, urothelial cancer*.

### Anti-VEGF Antibody and ICIs

When combined with chemotherapy, bevacizumab (an anti-VEGF monoclonal antibody) has been shown to prolong the survival of patients with cancer, especially for those with metastatic colorectal cancer (mCRC) and non-small cell lung cancer (NSCLC).

Bevacizumab, in combination with intravenous fluorouracil-based chemotherapy, is indicated for the first- or second-line treatment of patients with mCRC. A series of phase III clinical studies have demonstrated the survival benefit of adding bevacizumab to chemotherapy (74–76). The most common backbone regimen for combination is FOLFOX (fluorouracil–leucovorin–oxaliplatin) (77). The phase III AVEX study, comparing capecitabine–bevacizumab with capecitabine monotherapy in elderly patients (aged > 70 years) with untreated mCRC, showed a significant bevacizumab-related benefit in median progression-free survival (PFS) (78). A phase IV study (NCT01506167) provided evidence that there were no clear differences observed in outcomes between bevacizumab with capecitabine-based chemotherapy and fluorouracil-based regimens and confirmed the safety profile of bevacizumab in a real-world UK-based population (79).

Several meta-analyses have confirmed the benefit of bevacizumab in terms of PFS and OS in the first-line treatment of mCRC. However, a subgroup analysis suggested that the bevacizumab-related survival benefit is observed only when combined with irinotecan-based chemotherapy (80–83). Nevertheless, it is widely accepted that the addition of bevacizumab to first-line chemotherapy offers a modest clinical benefit.

The Center for Drug Evaluation of China approved bevacizumab, in combination with platinum-based doublet chemotherapy, for the first-line treatment of patients with unresectable, locally advanced, recurrent or metastatic non-squamous NSCLC. Adding bevacizumab to platinum-based chemotherapy in advanced non-squamous NSCLC confers a significant benefit in terms of PFS and OS (84–86) compared with chemotherapy alone.

In a meta-analysis, the addition of bevacizumab to chemotherapy can significantly improve PFS and overall response rate (ORR) both in first- and second-line treatments of advanced NSCLC; however, there was no benefit in terms of OS (87). In the setting of maintenance therapy, PFS was significantly improved by bevacizumab combined with pemetrexed following first-line induction vs. bevacizumab alone. However, OS was not prolonged upon combinatory maintenance therapy, and the treatment led to an increase in adverse events (AEs) (88, 89). Besides, the ERACLE phase III trial, using the evaluation of quality of life as the end point, illustrated that bevacizumab did not exert a superior effect on quality of life as maintenance therapy compared with pemetrexed (90). Some retrospective studies provided caveats regarding the benefit/risk profile in elderly patients (91–93). Therefore, bevacizumab in combination with chemotherapy is not recommended for eligible patients aged > 75 years. This subgroup of patients did not obtain significant benefit in terms of ORR, PFS, and OS from the addition of bevacizumab. Moreover, elderly patients in the bevacizumab group experienced a higher incidence of ≥ grade 3 AEs.

Bevacizumab is the first approved anti-VEGF monoclonal antibody that can maximize the clinical benefit of immunotherapy with ICIs. A phase I trial was designed to determine the safety and efficacy of bevacizumab in combination with the CTLA-4 inhibitor ipilimumab in metastatic melanoma (59). A total of 46 patients were included in that study and treated with four dosing combinations of ipilimumab (3 or 10 mg/kg) and bevacizumab (7.5 or 15 mg/kg). Eight and 22 patients had partial response (PR) and stable disease (SD), respectively. The disease control rate (DCR) was 67.4%. Median OS time was 25.1 months. Toxicities were generally higher than expected with ipilimumab alone but remained manageable. Eleven patients experienced grade 3 treatment-related adverse events (TRAEs), and two patients had grade 4 proteinuria and hepatic toxicities. Data from a randomized phase II clinical trial of patients with BRAF wild-type metastatic melanoma suggest an advantage with the sequential use of ipilimumab followed by nanoparticle albumin-bound-paclitaxel + bevacizumab (94). A randomized phase II trial (NCT01950390) to compare the OS of patients with unresectable stage III or stage IV melanoma receiving ipilimumab plus bevacizumab vs. ipilimumab monotherapy is currently ongoing. A phase II study (NCT04091217) evaluating the efficacy and safety of atezolizumab in combination with bevacizumab in patients with unresectable locally advanced or metastatic mucosal melanoma is currently in the patient recruitment phase.

In a retrospective study, 10 patients with untreated mRCC received a single dose of bevacizumab and subsequent combined administration of the anti-PD-L1 antibody atezolizumab and bevacizumab. A total of four patients achieved PR, while another four patients had SD. The median time to response was 4.2 months; however, the median duration of response was not reached (35). The phase II IMmotion150 study demonstrated that combination of bevacizumab and atezolizumab was superior to monotherapy with sunitinib in mRCC (95, 96). The phase III IMmotion151 study, including 915 untreated patients with mRCC, showed a prolonged PFS for PD-L1-positive patients after treatment with bevacizumab and atezolizumab vs. monotherapy with sunitinib (67). Furthermore, addition of bevacizumab to other PD-1/PD-L1 inhibitors (e.g., nivolumab or pembrolizumab) showed potent clinical activity in advanced and pretreated RCC (97, 98). The combination of bevacizumab and nivolumab had a higher ORR than that of nivolumab and ipilimumab (52 vs. 38%, respectively) (98).

In the phase III IMpower150 trials involving untreated patients with advanced non-squamous NSCLC, the combination of bevacizumab and platinum-based chemotherapy and atezolizumab improved PFS compared with chemotherapy and bevacizumab, regardless of the PD-L1 expression status (54). A phase I study evaluating maintenance therapy in patients after post-platinum doublet chemotherapy showed promising median PFS in non-squamous patients: 37.1 weeks with nivolumab and bevacizumab, and 21.4 weeks with nivolumab monotherapy (56). The safety profile was tolerable with nivolumab and bevacizumab. In the KEYNOTE-021 study, the median PFS in cohort B (pembrolizumab/carboplatin/paclitaxel/bevacizumab + pembrolizumab/bevacizumab maintenance) was not reached (NR) vs. that recorded at 10 months in cohort A (without bevacizumab) and cohort C (without bevacizumab) (55). Moreover, there were no pembrolizumab dose-limiting toxicities reported and the proportion of grade 3/4 TRAEs in cohort B was 42%. However, an estimation of efficacy cannot be affirmed because of the small numbers at the data cutoff (99).

For advanced HCC, a phase Ib study combining atezolizumab and bevacizumab confirmed that the response rate was 62%, suggesting that the combination therapy has synergistic clinical activity (61). Grade 3–4 TRAEs occurred in nine patients (35%), and grade 5 AEs were not observed. Given the safety and tolerability of this combination therapy, the US Food Drug Administration (FDA) approved atezolizumab + bevacizumab for the treatment of advanced HCC. During the 44th Congress of the European Society for Medical Oncology, the positive results of the IMbrave 150 phase III study (62) were inspiringly published, revealing a PFS of 6.8 months in the atezolizumab + bevacizumab arm vs. 4.3 months in the sorafenib arm; the ORR was 27 vs. 12%, respectively. At 2020 American Society of Clinical Oncology (ASCO) annual meeting, a network meta-analysis suggested greater OS and PFS benefits with first-line atezolizumab + bevacizumab over other therapies approved for unresectable HCC (100).

In a single practice case series including 20 patients with glioblastoma treated with the combination of ipilimumab and bevacizumab, Carter et al. (101) reported 31% PR, 31% SD, and 38% progressive disease. The toxicity profile of this regimen was basically predictable and manageable. Grade ≥ 3 AEs were found in seven patients (35%), and only two patients discontinued treatment due to AEs (10%).

### Anti-VEGFR Antibody and ICIs

Anti-VEGFR antibody also showed a synergistic anti-cancer effect when combined with a checkpoint inhibitor. In a phase I trial of patients with advanced gastric or gastroesophageal junction adenocarcinoma (64), ramucirumab in combination with pembrolizumab led to a promising ORR (7 and 17%) and DCR (46 and 50%) in pretreated and untreated patients, respectively. This combination effect was also confirmed in advanced NSCLC (57, 102). Of the 27 patients with NSCLC included in the study, 30% had an objective response and 85% experienced disease control. Median PFS was not reached, and the median duration of treatment was ≥ 6.8 months. In the pretreated group (59% had received at least two lines of therapy), these results were encouraging. However, the small number of patients limited the confirmation of the effectiveness of this combination against NSCLC. The efficacy of this combination was also observed in urothelial cancer (103).

In an early-stage trial (NCT02572687), the efficacy of the combination of ramucirumab and durvalumab in gastric or gastroesophageal junction carcinomas was reported; five of 29 patients (17%) achieved PR, the ORR for patients with PD-L1 ≥ 25% was 36%, and the median PFS was 2.6 months (65). TRAEs were observed in 24 patients (83%) and 10 patients (35%) suffered grade 3 TRAEs without occurrence of grade 4 or 5 TRAEs.

### VEGFR TKIs and ICIs

The combination of VEGFR TKIs with ICIs in RCC, HCC, NSCLC, mucosal melanoma, endometrial carcinoma, esophageal carcinoma, triple-negative breast cancer, microsatellite stability (MSS) gastric carcinoma (GC) and CRC, head and neck squamous cell carcinoma, urothelial carcinoma, osteosarcoma, and other malignant tumors has been associated with favorable outcomes (104).

A group of different VEGFR TKIs were tested in combination with PD-1 blockade in mRCC. The efficacy of axitinib in combination with pembrolizumab in patients with advanced RCC was preliminarily investigated in a phase Ib trial. Following the combination therapy, the ORR was 73%, the proportion of patients achieving complete response was 8%, and tumor shrinkage occurred in > 90% of patients. Only two patients failed to attain tumor shrinkage or stable disease, with a median PFS of > 20 months vs. 10–15 months in the axitinib monotherapy group (105–107). A phase II study following the phase Ib trial of lenvatinib + pembrolizumab in patients with mRCC manifested promising antitumor activity, including a median PFS of 17.7 months [95% confidence interval [CI]: 11.9–15.7] and ORR of 66.7% (95% CI: 43.9–80.1) (70) compared with the confirmed ORR of 40.4% for the combination of nivolumab and ipilimumab in mRCC (108, 109). Additionally, nivolumab in combination with sunitinib or pazopanib in patients with mRCC (CheckMate 016 study) that had received more than one prior systematic treatment showed encouraging outcome. The ORR was 55% (18/33) and 45% (9/20) in the sunitinib and pazopanib arms, respectively. Meanwhile, rate of response at the first assessment (week 6) was 41% (sunitinib arm) and 56% (pazopanib arm) (72). However, the scheme of nivolumab plus sunitinib or pazopanib were not recommended due to the high incidence (82 and 70%) of grade 3–4 TRAEs. In 2018, Choueiri et al. (68) reported the efficacy of avelumab and axitinib combination therapy in treatment-naive patients with advanced clear cell RCC (NCT02493751). During nearly 1 year of follow-up, the OS in the combination therapy was 58% (32/55), while the rate of SD was 20% (11/55) (68). In this study, it was observed that the diverse expression levels of PD-L1 did not significantly affect the efficacy of treatment. Motivated by the preliminarily encouraging results of NCT02493751, a phase III clinical trial (NCT02684006) (JAVELIN Renal 101) aiming to compare the efficacy of avelumab plus axitinib vs. monotherapy with sunitinib in advanced clear cell RCC was sequentially conducted. Median PFS in patients with PD-L1+ tumors receiving avelumab plus axitinib was not estimable (8.1 months, not estimable), while that noted in patients receiving sunitinib was 11.2 months; in patients irrespective of PD-L1 expression, the median PFS was 16.6 months vs. 11.2 months, respectively. The ORR was 60.6 vs. 17.6%, respectively. Common TRAEs (grade ≥ 3) in each group were hand-foot syndrome (9 vs. 9%, respectively), hypertension (30 vs. 18%, respectively), and platelet count decreased (0 vs. 32%, respectively) (69). Given the bracing results of JAVELIN Renal 101 study, avelumab combined with axitinib was approved by FDA. In a phase III KEYNOTE-426 study investigating pembrolizumab plus axitinib vs. sunitinib as first-line therapy for advanced RCC, the median PFS and ORR in the pembrolizumab plus axitinib arm vs. the sunitinib arm were 15.1 months vs. 11.1 months and 59.3 vs. 35.7%, respectively. Grade ≥ 3 AEs occurred in 75.8% of patients in the pembrolizumab plus axitinib arm and in 70.6% of patients in the sunitinib arm (71). Based on this outcome, first-line therapy of pembrolizumab plus axitinib was approved for advanced RCC. Furthermore, the updated analysis of the phase III KEYNOTE-426 study presented that the combination continued to demonstrate superior and durable antitumor activity over sunitinib after a 27-months median follow-up and no new safety signals were observed (110). A multicenter, randomized, open-label, phase III study (NCT02811861) to compare the efficacy and safety of lenvatinib in combination with everolimus or pembrolizumab vs. sunitinib as first-line treatment in participants with advanced RCC is ongoing.

In the field of combination therapies for advanced HCC, SHR-1210 (known as camrelizumab, an anti-PD-1 antibody manufactured in China) in combination with apatinib yielded promising results: ORR of 30.8% (95% CI: 17.0–47.6) and PR rate of 50.0% (95% CI: 24.7–75.4) (60). Of note, this combination therapy offered a statistically significant advantage for patients with advanced HCC over the previous clinical trial of monotherapy with nivolumab (111). A phase Ib study assessing the combination of pembrolizumab and lenvatinib treating patients with unresectable HCC was revealed at 2020 ASCO annual meeting. Median OS was 22.0 months (95% CI: 20.4-NR), median PFS was 8.6 months (95% CI: 7.1–9.7), and ORR was 36% (95% CI: 26.6–46.2), and the safety profile was tolerable (63). Meanwhile, a real-world study about unresectable HCC in Taiwan demonstrated pembrolizumab plus lenvatinib can produce excellent ORR and DCR with tolerable safety profiles (112). In a study using an animal model of HCC, Kimura et al. (113) highlighted that lenvatinib has immunoregulation activity in mice, which contributes to its antitumor activity. This activity also enhances the antitumor activity of anti-PD-1 antibody in combination therapy (113). Currently, multiple clinical trials in different phases are underway, intending to reveal the role of combination therapy based on ICIs plus VEGFR TKIs in treating advanced HCC. For example, a large phase III trial aims to evaluate the efficacy of the combination of lenvatinib plus pembrolizumab as a first-line therapy (NCT03713593), while a phase I trial was designed to assess the efficacy of a combination of lenvatinib with nivolumab (NCT03418922).

Han et al. (58) reported a similar evolution in patients with advanced NSCLC. The first clinical trial to assess the efficacy and safety of sintilimab with anlotinib as first-line therapy has indicated a synergistic effect against advanced NSCLC. Sixteen patients (72.7%) achieved PR and six patients (27.3%) achieved SD with an ORR of 72.7% and a DCR of 100%; the 6-month PFS rate was 93.8%. Notably, five of the six patients with SD had cavities in tumors, manifesting that the combination regimen exhibits synergy. Grade ≥ 3 TRAEs occurred in six patients (27.3%) and remained manageable.

Sheng et al. (114, 115) conducted an open-label, phase Ib trial combining axitinib with toripalimab (the third approved anti-PD-1 antibody manufactured in China) in patients with metastatic mucosal melanoma. Among 29 chemotherapy-naïve patients with mucosal melanoma, the ORR was 48.3% (*n* = 14, 95% CI: 29.4–67.5), the median PFS was 7.5 months (95% CI: 3.7–NR) and the median OS was 20.7 months. The vast majority (97%) of patients experienced TRAEs, with mild (grade 1 or 2) TRAEs being the most common; 39.4% of patients experienced grade ≥ 3 TRAEs.

The results of a phase Ib/II study applying combination therapy of pembrolizumab and lenvatinib in patients with advanced endometrial cancer were revealed at the 2018 ASCO annual meeting (73). A total of 53 patients with advanced endometrial cancer were included in the study. The ORR was 39.6% (21/53, including three patients with complete response) and the median PFS was 7.4 months. The incidence of grade 3 TRAEs was 59%, and there was no occurrence of grade 4 TRAEs. Of note, in this study, the efficacy of the combination therapy was not associated with some common important predictive biomarkers, such as microsatellite instability and PD-L1. Moreover, a study of pembrolizumab plus lenvatinib for early-line treatment of previously treated, advanced, non high-frequency microsatellite instability (MSI-H) or mismatch-repair deficiency (dMMR) endometrial cancer patients was reported at the 2020 ASCO annual meeting (116). Patients were divided in two subgroups, the ORR was 41.3% (*n* = 63, 95% CI, 29.0–54.4) for subgroup 1 and 57.1% (*n* = 21, 95% CI, 34.0–78.2) for subgroup 2. In subgroup 1, 42 (67%) of patients were exposed to grade ≥ 3 TRAEs, serious TRAEs occurred in 18 (29%) patients and 2 (3%) patients died from TRAEs. The safety profile for subgroup 2 was generally similar to that for subgroup 1.

The REGONIVO study included 50 patients with advanced GC or CRC, with a median of three lines of previous treatment, who received regorafenib plus nivolumab (117). Accordingly, the ORR was 40%, the DCR was 88%, and the median duration of treatment was 6.1 months. In the CRC group, the general ORR and median PFS were 36% and 6.3 months, respectively; the ORR of patients with MSS CRC was 33%. All examined GC cases were of the MSS type; the ORR was 44% and the median PFS was 5.8 months. The outcome of a phase II trial assessing the efficacy and safety of regorafenib + avelumab combination in non MSI-H metastatic CRC patients has been released recently (66). The combination achieved better PFS and OS compared to regorafenib alone with historical data of in this clinical setting. In this trial, median number of previous treatment lines was 3 (range: 1–7). The median PFS and OS were 3.6 months (95% CI: 1.8–5.4) and 10.8 months (95% CI: 5.9-NR), respectively, and the safety profile was manageable. It was also reported that high infiltration by TAMs at baseline was significantly associated with adverse outcome (PFS: 1.9 vs. 3.7 months, *p* = 0.045; OS: 4.8 months vs. NR, *p* = 0.027) and increased tumor infiltration by CD8+ T cells compared to baseline was significantly associated with better PFS (*p* = 0.011). Besides, the efficiency of fruquintinib (a VEGFR inhibitor manufactured in China) combined with sintilimab (an anti-PD-1 antibody manufactured in China) in refractory metastatic CRC patients in China was evaluated (118). Median PFS was 108 days, whereas it seemed not resulted in a significant increase in ORR, DCR and OS. In the evaluation, patients with PFS <90 days was considered worse subgroup, and they were found have the following mutations: AMER1 (*p* = 0.0073), DNMT3A (*p* = 0.0075), ETV5 (*p* = 0.012), EWSR1 (*p* = 0.016), FANCA (*p* = 0.019), IKBKE (*p* = 0.0073), NOTCH1 (*p* = 0.015), STAG2 (*p* = 0.012), and TCF7L2 (*p* = 0.0073), which suggested targeting these mutational genes may be helpful to improve the efficacy.

## Predictive Indicators for Combination Therapy of Anti-Angiogenic Agents and ICIs

The indication of anti-angiogenesis TKIs is mainly restricted to highly vascular tumors, such as RCC, HCC, NSCLC, endometrial cancer, and CRC (119). A barrier for the use of these TKIs is the lack of sensitive and valid biomarkers. VEGF-A121, a secreted isoform of the VEGF-A family (VEGF-A121, VEGF-A165, VEGF-A189, and VEGF-A206) (120), has been intensively studied as a measurable biomarker predicting the efficacy of VEGF targeted agents (121). Unfortunately, it failed to yield satisfactory results as a potent predictive biomarker. Measurement of the tumor vascular function through dynamic contrast-enhanced magnetic resonance imaging (122) and the baseline levels of IL-6 (123, 124) were also declared predictors of PFS and OS benefit in patients with RCC after pazopanib therapy. Other biomarkers were successively proposed, such as VEGFR-2, fibroblast growth factor 2 (FGF-2), or IL-8. However, their use has not been established in routine clinical practice (125). Recently, a cohort study indicated a positive correlation between the anti-angiogenesis-related AEs and prolonged OS (126, 127). Similarly, several investigations demonstrated that anti-angiogenesis-related hypertension can be considered a predictor of OS and PFS benefit after treatment with VEGF TKIs (128, 129). Considering the lack of a robust biomarker for routine clinical use, TRAEs may be helpful in predicting efficacy.

For ICIs, some predictive biomarkers have been strongly associated with the response to such therapy with monoclonal antibodies. These include, PD-L1 in NSCLC and advanced urothelial carcinoma patients treated with pembrolizumab (130, 131), tumor mutation burden (132), and MSI-H/dMMR in patients with pembrolizumab for unresectable or metastatic mismatch-repair deficient solid tumors (133). Multiple studies have proposed the absolute lymphocyte count and lactate dehydrogenase in peripheral blood as potential predictive biomarkers. These data suggested that an increase in absolute lymphocyte count or the levels of lactate dehydrogenase predicted more positive response of patients (134–136). The KEYNOTE-086 study (137) used lymph nodes and cutaneous/subcutaneous metastatic surgical samples resected from patients with metastatic melanoma treated with ipilimumab to validate the presence of tumor-infiltrating lymphocytes. The results showed that, especially CD16+ and CD68+ cells, were associated with an affirmative response to ICIs, as well as prolonged survival. Subsequent studies including patients with NSCLC or metastatic melanoma receiving ICIs discovered that neoantigens coded by DNA with loss-of-function mutations can result in drug resistance (138–140). Alterations in PD-L1 and PD-L2 copy number (141), as well as microsatellite instability/mismatch-repair deficiency (142) were identified as potential predictive biomarkers. Lately, the ratio of metabolic to morphological lesion volumes for patients with NSCLC (143) and tumor heterogeneity index for patients with metastatic melanoma (144), calculated through 2-deoxy-2-(18F)fluoro-D-glucose positron-emission tomography/computed tomography, have been utilized as imaging biomarkers for ICI therapy.

Based on the successful combination of anti-angiogenic agents with ICIs and the ongoing research, it becomes clear that the use of a single predictive biomarker regardless of cancer type or combination therapy regimen may be inappropriate (59). Wallin et al. (35) confirmed that the combination of bevacizumab and atezolizumab in mRCC promotes antigen-specific T-cell migration. Meanwhile, elevation of intra-tumoral MHC-I, Th1, and T-effector markers, and chemokines (most notably CX3CL1) was also found. Moreover, the VEGF inhibitor improves the levels of the CX3CL1 receptor CX3CR1 on periphery CD8+ T cells (145). In the IMmotion 150 and IMmotion 151 trials comparing bevacizumab + atezolizumab vs. monotherapy with sunitinib in mRCC, patients with tumors with T-effector/IFN-γ-high response or high myeloid inflammatory gene expression signatures had better PFS after treatment with the combination regimen (146, 147). Remarkably, the IMpower150 study addressed that atezolizumab + bevacizumab benefitted the EGFR or ALK wild-type patients with high expression of T-effector gene signature, which substituted the PD-L1+ populations in defining the other group for primary endpoint analysis (148).

The synergetic promotion of the intra-tumoral chemokine and its receptor in the periphery implies the presence of a lymphocyte-trafficking mechanism associated with inhibition of VEGF. In a phase I clinical trial (NCT00790010) to investigate the effect of ipilimumab plus bevacizumab in patients with metastatic melanoma, patients receiving the combination regimen showed a great advantage in prognosis (median OS, combination therapy vs. ipilimumab monotherapy: 25.1 vs. 10.1 months, respectively) (59, 149). In addition, conspicuous upregulation of CD31, E-selectin, VCAM-1, and other adhesion molecules on intra-tumoral endothelia cells were recorded (150). In a clinical trial investigating the combination therapy of camrelizumab plus apatinib, the efficacy in patients with GC/esophagogastric junction cancer was unsatisfactory, although the therapeutic effect in patients with HCC was encouraging. We can presume that the discrepant consequences among these different types of cancers may be attributed to tumor immunogenicity (60). Interestingly, in numerous studies, elevation in PD-L1 expression is considered a consequence of hypoxia induced by anti-angiogenic treatment (151) or independent of hypoxia or hypoxia-inducible factor 1α (HIF1α) (152, 153). This implies that the detection of PD-L1 expression is not applicable for prediction during treatment with this combination therapy. The JAVELIN Renal 101 study demonstrated that the PD-L1 expression levels cannot distinguish whether the combination therapy benefits PFS, while the high levels of intratumoral CD8+ T cells were strongly associated with better efficacy. Notably, 26 gene profiles, including immune-related genes, may be potential biomarkers for this combination (69).

In summary, numerous predictive biomarkers with diverse functions have been studied in anti-angiogenesis and immune checkpoint blockade, respectively. However, multiple sensitive and efficient predictors for anti-angiogenic agents in combination with ICIs are currently being investigated, where both cancer type and combination therapy regimen should be taken into consideration. As the authors are concerned, it seems rare difficult to determine a common indicator applied to several kinds of cancer, probably owing to different tumor immunogenicity resulting in distinct response to various medicines. Another reason is assumed to be the objective lack of relevant clinical trials. Since this combination therapy is emerging and the antibodies/TKIs are rapidly developing, the amount of certain trials is increasing at a low baseline and the data of detecting biomarkers is insufficient. Thus far, no appropriate predictive biomarker has been identified and clinical trials need accumulate more.

## Safety of Combination Therapy of Anti-Angiogenic Agents and ICIs

Undoubtedly, both types of therapy will result in complicated biological responses. This complexity was further enhanced since the development of the combination strategy, suggesting that an increased risk of toxicities may ensue. In a systematic review (154), it was clarified that the risk of cardiovascular events, hypertension, arterial thromboembolism, and proteinuria associated with anti-angiogenic agents was high. Meanwhile, immune-related AEs, such as autoimmune colitis (155), immune-related pneumonitis (156), and immune-related dermatitis (157) are relatively frequent symptoms. According to previous studies, toxicities (both anti-angiogenesis-related and immune-related AEs) have been simultaneously or, respectively, observed during treatment in the majority of cases. The synergistic effects of combination treatment are affirmative. However, theoretically, the frequencies and degrees of the two aforementioned toxicities may be elevated accordingly (158). In a former study combining pazopanib and nivolumab in the treatment of mRCC, the addition of nivolumab enhanced the pazopanib-related unpredictable elevation of transaminases (159). In contrast, based on the clinical data available thus far, there is no appearance of novel anti-angiogenesis-related or immune-related toxicity, and the toxicity profile of the regimen remains tolerable (with only the spectrum of TRAEs expanding). The evaluation of the safety of ICIs in combination with anti-angiogenic agents demands consideration of both the tumor type and position, requiring further investigation. Reports have shown that treatment with ICIs increases the risk of edema in brain parenchyma for patients with primary brain tumors and metastatic encephaloma, and my even result in death (160, 161). Nevertheless, anti-VEGF agents are reported to decrease the risk of glioblastoma-associated brain edema in both mice and patients (162, 163), offering promise for the combination of anti-VEGF therapy and ICIs in the treatment of glioblastoma and possibly brain metastases. In breast cancer models, a vascular normalization effect induced by ICIs was observed (34), contrasting to the severe cerebrovascular events noted in former cases. Toxicities caused by ICIs can often resolve after discontinuing or reducing the treatment doses (155). Considering that vascular normalization due to the anti-angiogenic agents can improve the delivery of therapeutic agents to tumors (164), the proposed combination strategy may not require large doses of ICIs to maintain its immunostimulatory effect and simultaneously reduce the risk of immune-related AEs (2). In the CheckMate 016 study, higher frequencies of high-grade (3 or 4) TRAEs were reported with combination therapy than monotherapy with nivolumab, sunitinib, or pazopanib. In addition, the frequencies of AEs resulting in treatment discontinuation were higher with the combination regimen than monotherapy with nivolumab, sunitinib, or pazopanib. Notably, responses to nivolumab combined with sunitinib or pazopanib and the OS outcome were encouraging. When compared with other regimens of combination therapy, the discoveries of the CheckMate 016 study suggest that the safety and efficacy of different regimens based on ICIs combined with anti-angiogenic agents may depend on thoughtful selection of the anti-angiogenic component and dose. Of note, the accurate mechanism of TRAEs occurrence during this treatment (anti-angiogenesis-related or immune-related) can differ owing to individual differences. For instance, both drugs may lead to thrombocytopenia, which may be a consequence of anti-angiogenic agent-induced thrombotic microangiopathy and atypical hemolytic uremic syndrome or ICIs-induced overactivation of T cells (165–167). According to those reports, the treatment-related thrombocytopenia diminished with the discontinuation of agents or intravenous monoclonal antibodies (e.g., eculizumab) from the perspective of anti-angiogenesis therapy, or with the application of corticosteroids or immunosuppressive drugs from the perspective of ICIs therapy. Hence, clinicians are requested to distinguish the pathogenesis for adopting optimal countermeasures.

With ICIs attracting more attention in the treatment of patients with HCC, several clinical trials combining ICIs with anti-angiogenic agents are currently recruiting patients with HCC worldwide. For example, a phase III, randomized, active-controlled trial aiming to assess the safety and efficacy of lenvatinib in combination with pembrolizumab compared with lenvatinib plus placebo as first-line therapy of advanced HCC (NCT03713593) is ongoing. Moreover, a phase II trial evaluating sorafenib combined with nivolumab as first-line therapy is also in the recruitment phase (NCT03439891).

Overall, the toxicity of anti-angiogenesis therapy combined with ICIs is not severe, and lower than that of ICIs combined with chemotherapy. Most existing clinical trials show tolerable or manageable safety profile. In authors' opinion, the doses of ICIs or anti-angiogenic agents can be slightly inadequate to reduce the degree and frequency of TRAEs, and the administration time of each agent during the treatment is worth considering to achieve better efficacy. However, physicians are required to carefully identify the source of the TRAEs associated with this combination to precisely determine the corresponding countermeasures.

## Conclusion

Although immunotherapy with ICIs in the treatment of cancer is undoubtedly one of the most promising strategies, serious challenges remain (e.g., low response rate, AEs, and acquired resistance). Studies have revealed that anti-angiogenesis and ICIs therapy can reprogram the tumor milieu from an immunosuppressive to an immune permissive microenvironment, and mutually enhance the antitumor effect. Firstly, deeper comprehension of the impact anti-angiogenic agents and immunotherapy have on the immune system of patients with cancer, and the mechanism of mutual enhancement is warranted. Secondly, diverse combination therapy regimens involving ICIs (PD-1, PD-L1, and CTLA-4 inhibitors) combined with anti-VEGF antibody, anti-VEGFR antibody, or VEGFR TKIs have shown more clinical benefit than ICIs or anti-angiogenic monotherapy and homogeneous combination therapy, providing a hopeful solution to the dilemma of immunotherapy with ICIs. Nevertheless, the timing or the sequence of each agent in the combination and the optimal regimen are currently unclear, while the optimal dose of each agent remains unknown. Thirdly, owing to the vascular normalization caused by anti-angiogenic agents, the delivery of therapeutic agents to tumors is improved, thereby reducing the doses of ICIs and decreasing the risk of immune-related AEs. Despite these exciting achievements, several safety problems urgently need to be resolved. Finally, the lack of sensitive and efficient predictive biomarkers for anti-angiogenic agents in combination with ICIs impedes the adjustment of the scheme on certain conditions. On the other hand, the evaluation of either safety or prognosis should take both the type of cancer and the selection of drugs into sensible consideration. This may lead to a low efficiency clinical decision and demand for a number of relevant studies in the future.

In conclusion, anti-angiogenic agents in combination with ICIs have demonstrated promising outcome in certain types of carcinoma. However, further intensive studies are warranted to resolve the above problems. The effectiveness, toxicity, and tolerability of the combination therapy need to be optimized by determining the appropriate dose and sequence. Anti-angiogenic agents combined with ICIs are more suitable for patients with advanced malignant tumors who are not sensitive to, willing, or able to tolerate chemotherapy. This therapy has been proved competent for the treatment of HCC and RCC, but further research is still warranted to claim that the treatment of cancer has reached the chemotherapy-free era. In the future, basic researches investigating the mechanism of the positive feedback loop between anti-angiogenic agents and ICIs should be conducted in a more detailed and interconnected manner to help develop new formulation and design clinical studies. Meanwhile, not only the efficient of certain regimen is evaluated, biomarkers are supposed to be detected synchronously in more clinical trials. With increasing valid evidence, physicians are tending able to decide better combination and administration time and sequence, enhancing the efficacy and reducing the toxicities. Optimizing this new strategy into the most standard therapy requires a long distance.

## Author Contributions

BZha and JW provided access to the data and writing materials. YS and YF contributed to writing this manuscript. All authors made substantial contributions, read, and approved the final manuscript.

## Conflict of Interest

The authors declare that the research was conducted in the absence of any commercial or financial relationships that could be construed as a potential conflict of interest.
